# Supplementary material for: Ancestral male recombination in Drosophila albomicans produced geographically restricted neo-Y chromosome haplotypes varying in age and onset of decay
Source: PLoS Genet. 2019 Nov 18;15(11):e1008502. doi: 10.1371/journal.pgen.1008502 (PMC6897423; doi:10.1371/journal.pgen.1008502)
Supplement: S1 Fig — Strains are color coded based on their neo-Y type. (PDF) [file pgen.1008502.s005.pdf]

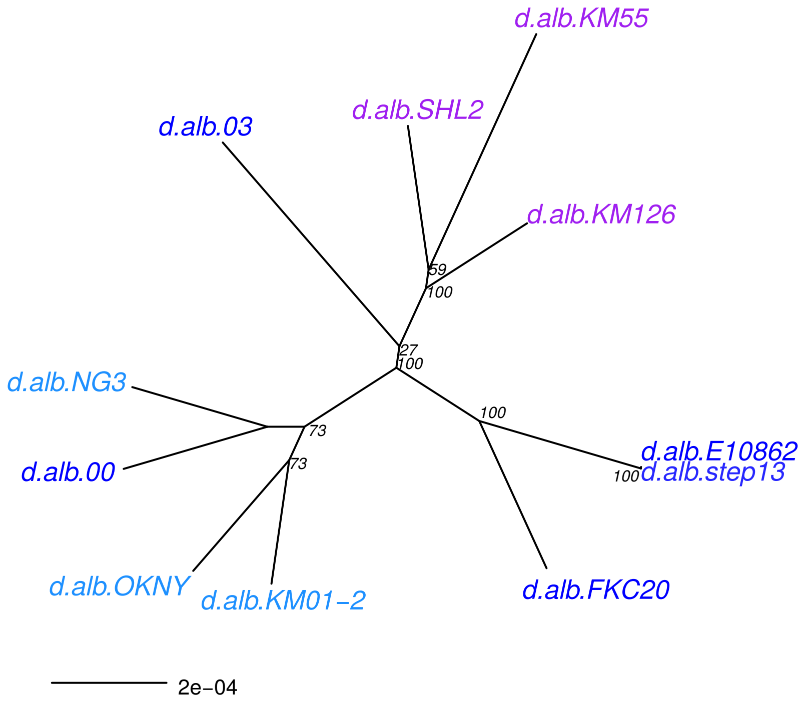

S1 Fig. The neo-X phylogeny of strains with neo-Y genotypes. Strains are color coded based on their neo-Y type.
