# Supplementary figures and images for: Ancestral male recombination in Drosophila albomicans produced geographically restricted neo-Y chromosome haplotypes varying in age and onset of decay
Source: PLoS Genet. 2019 Nov 18;15(11):e1008502. doi: 10.1371/journal.pgen.1008502 (PMC6897423; doi:10.1371/journal.pgen.1008502)

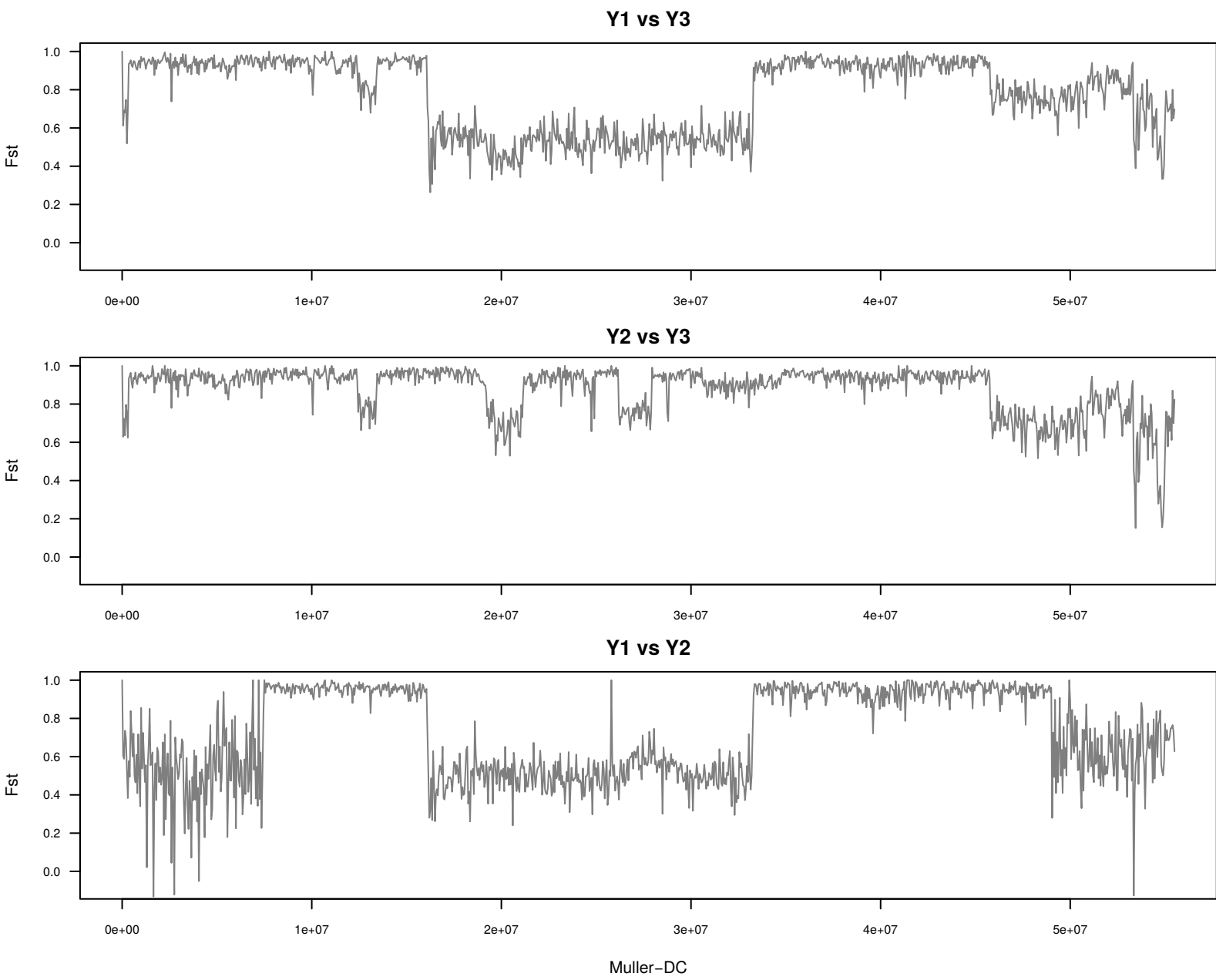

**S2 Fig.**  $F_{ST}$  between different Y populations

Supplement: S2 Fig — (PDF) [file pgen.1008502.s006.pdf]

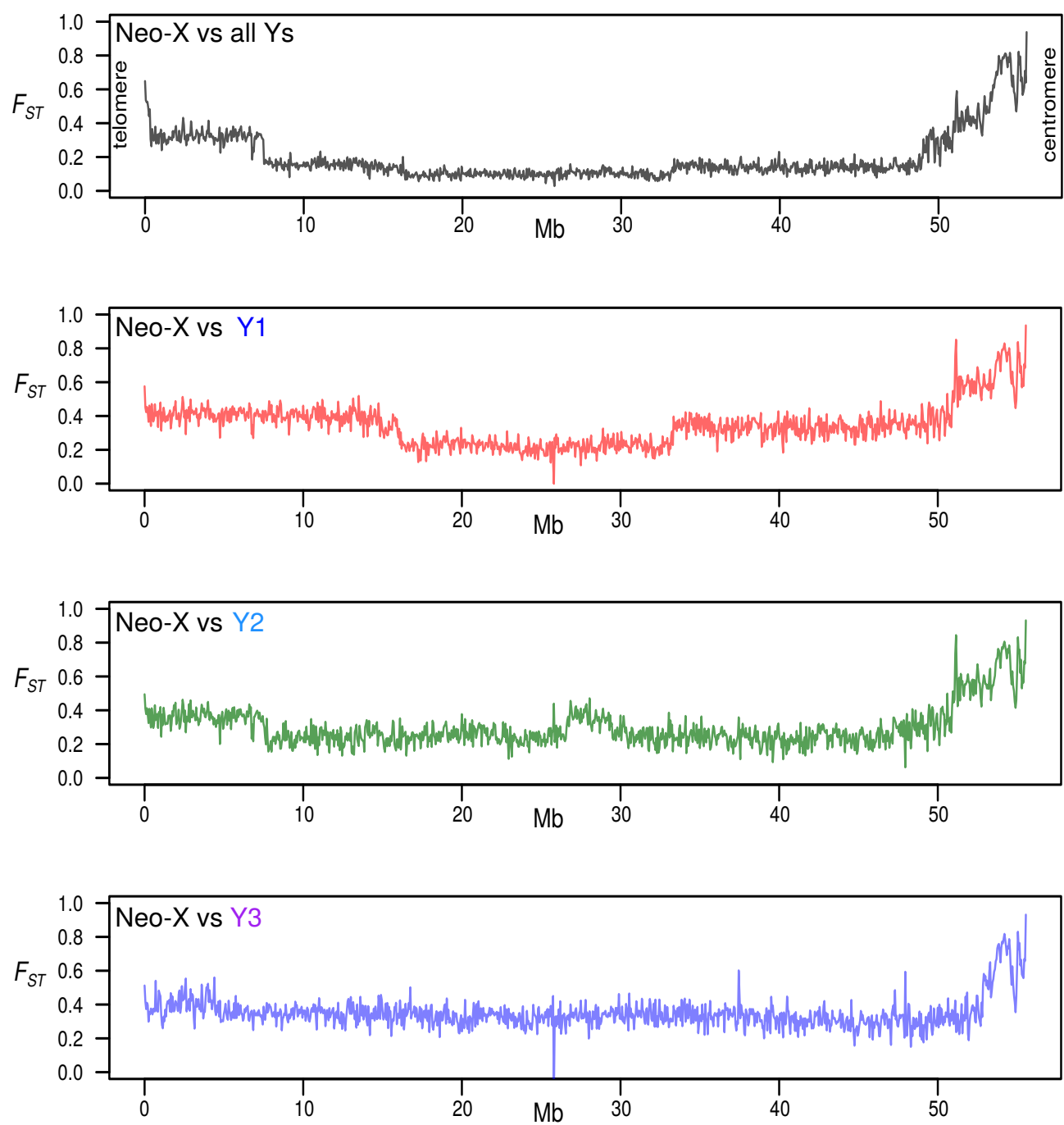

**S3 Fig.**  $F_{ST}$  between different Y populations and neo-X

Supplement: S3 Fig — (PDF) [file pgen.1008502.s007.pdf]

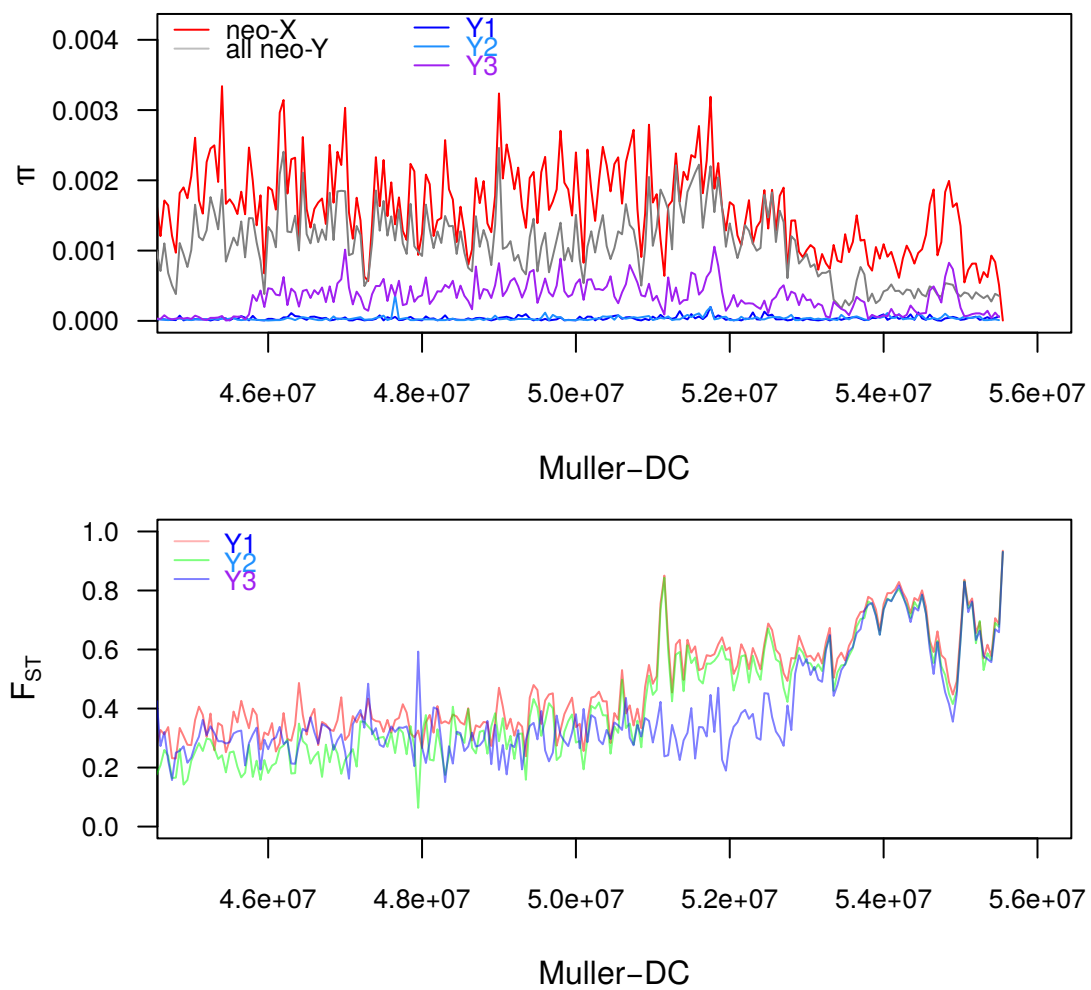

**S4 Fig.**  $F_{ST}$  and  $\pi$  at the centromere proximal region.

Supplement: S4 Fig — (PDF) [file pgen.1008502.s008.pdf]

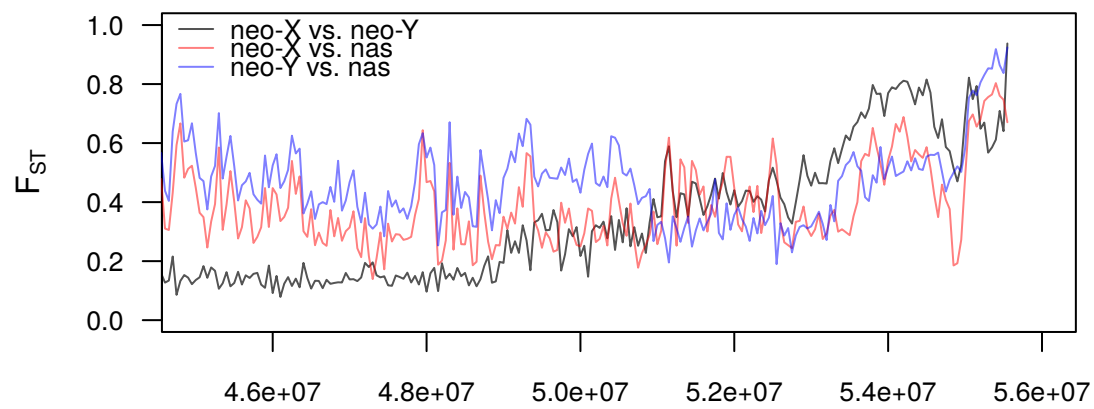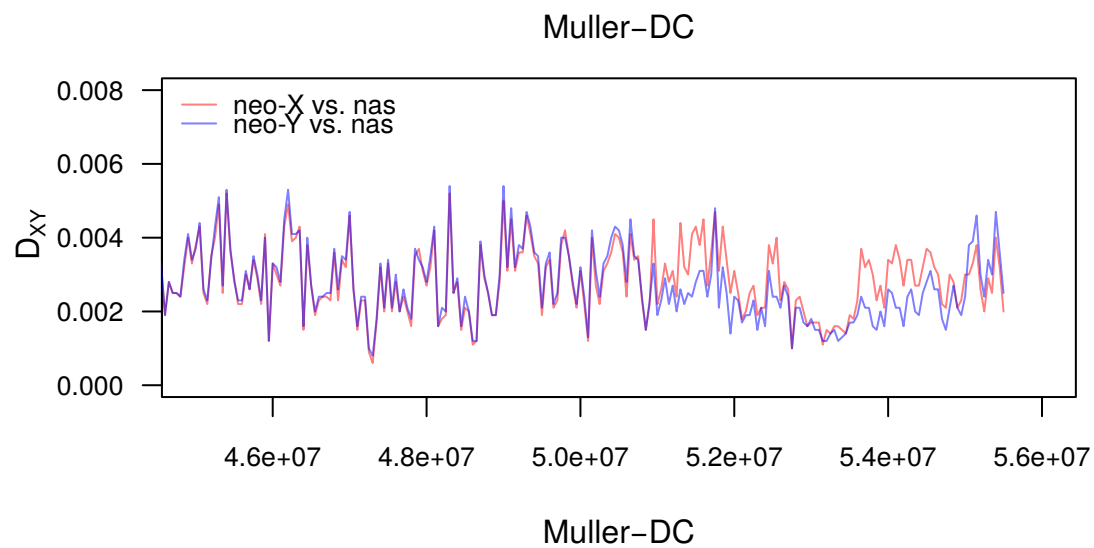

**S6 Fig.**  $F_{ST}$  and  $D_{XY}$  between the neo-X, neo-Y, and Chr. 3 the centromere proximal region.

Supplement: S6 Fig — (PDF) [file pgen.1008502.s010.pdf]
