# Supplementary material for: Ancestral male recombination in Drosophila albomicans produced geographically restricted neo-Y chromosome haplotypes varying in age and onset of decay
Source: PLoS Genet. 2019 Nov 18;15(11):e1008502. doi: 10.1371/journal.pgen.1008502 (PMC6897423; doi:10.1371/journal.pgen.1008502)
Supplement: S5 Fig — (PDF) [file pgen.1008502.s009.pdf]

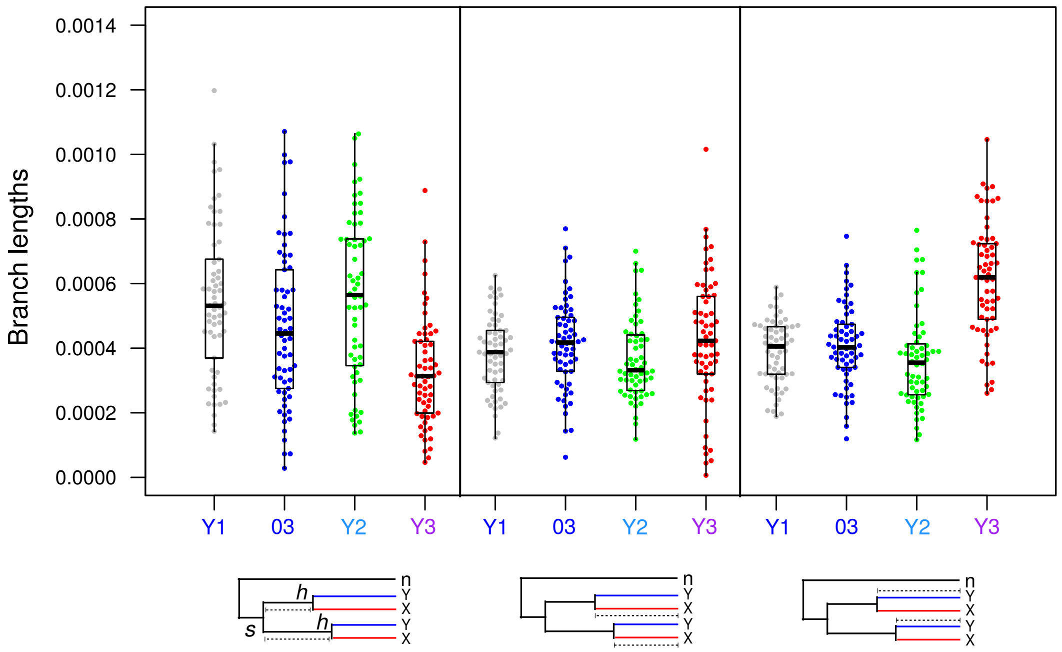

S5 Fig. Same as Figure 3C but estimates are restricted to windows between 16.2 Mb and 33.2 Mb, where all Y types have a different haplotype.
