# Supplementary material for: Ancestral male recombination in Drosophila albomicans produced geographically restricted neo-Y chromosome haplotypes varying in age and onset of decay
Source: PLoS Genet. 2019 Nov 18;15(11):e1008502. doi: 10.1371/journal.pgen.1008502 (PMC6897423; doi:10.1371/journal.pgen.1008502)
Supplement: S7 Fig — Windows where SHL-2 falls in the D. albomcians neo-X clade are colored red. Windows where SHL-2 falls in the D. nasuta Chr.3 clade are colored in yellow. (PDF) [file pgen.1008502.s011.pdf]

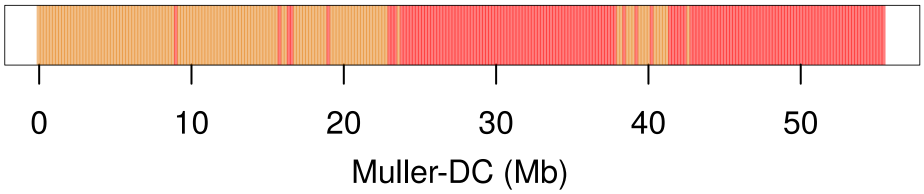

S7 Fig. *D. nasuta* introgression on the neo-X of SHL-2. Windows where SHL-2 falls in the D. albomicans neo-X clade are colored red. Windows where SHL-2 falls in the D. nasuta Chr. 3 clade are colored in yellow.
