# Supplementary material for: Ancestral male recombination in Drosophila albomicans produced geographically restricted neo-Y chromosome haplotypes varying in age and onset of decay
Source: PLoS Genet. 2019 Nov 18;15(11):e1008502. doi: 10.1371/journal.pgen.1008502 (PMC6897423; doi:10.1371/journal.pgen.1008502)
Supplement: S8 Fig — (PDF) [file pgen.1008502.s012.pdf]

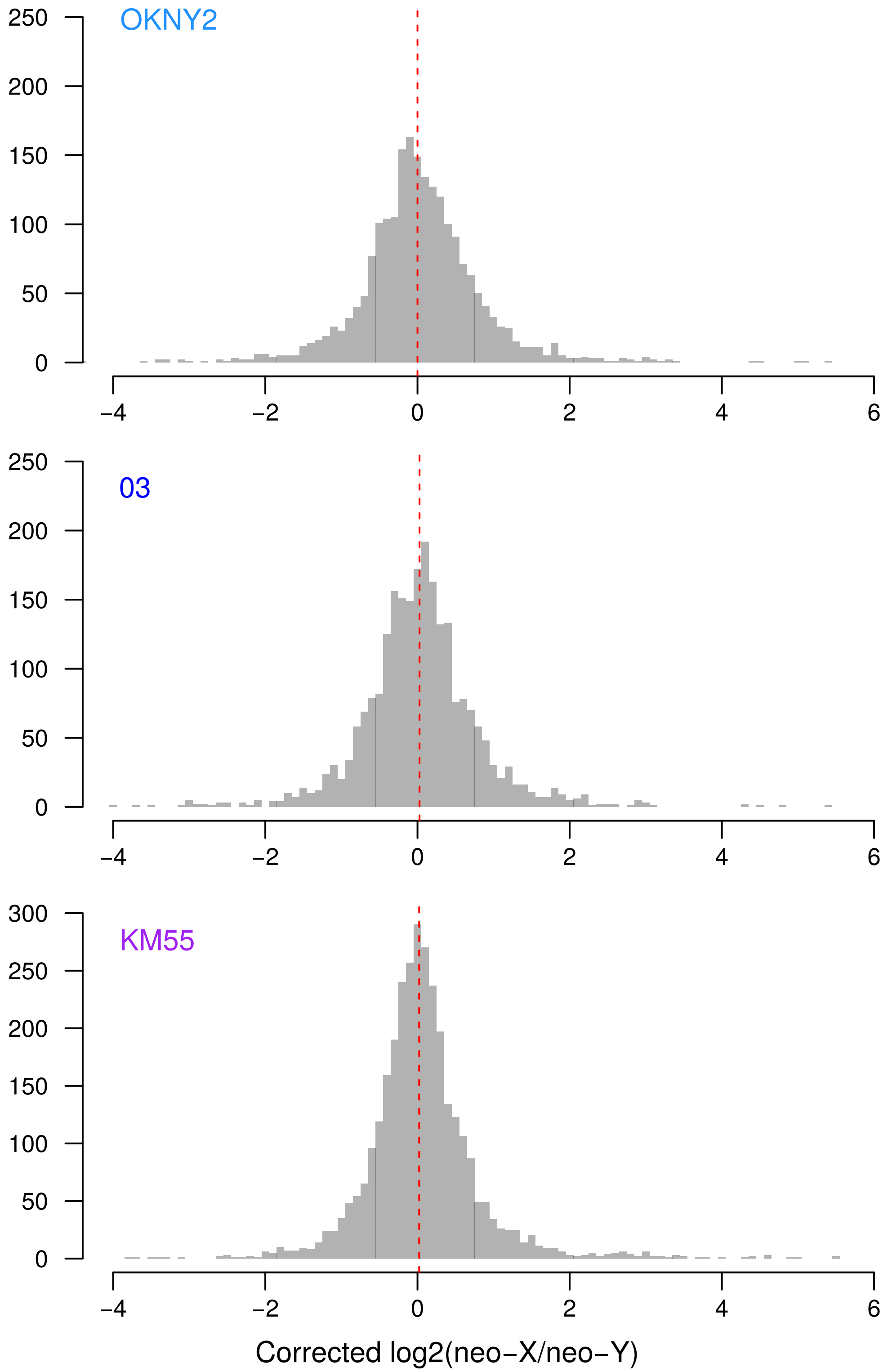

S8 Fig. Distribution of allele-specific gene expression across neo-Y chromosomes of different ages.
