# Supplementary material for: Ancestral male recombination in Drosophila albomicans produced geographically restricted neo-Y chromosome haplotypes varying in age and onset of decay
Source: PLoS Genet. 2019 Nov 18;15(11):e1008502. doi: 10.1371/journal.pgen.1008502 (PMC6897423; doi:10.1371/journal.pgen.1008502)
Supplement: S9 Fig — Left panels, allele-specific read counts over strain-specific SNP sites (points) differentiating the neo-X and neo-Y chromosomes were used to calculate the fold difference. Right panels, histograms of the distribution of the log2 fold differences. Red lines demarcate the median fold difference. (PDF) [file pgen.1008502.s013.pdf]

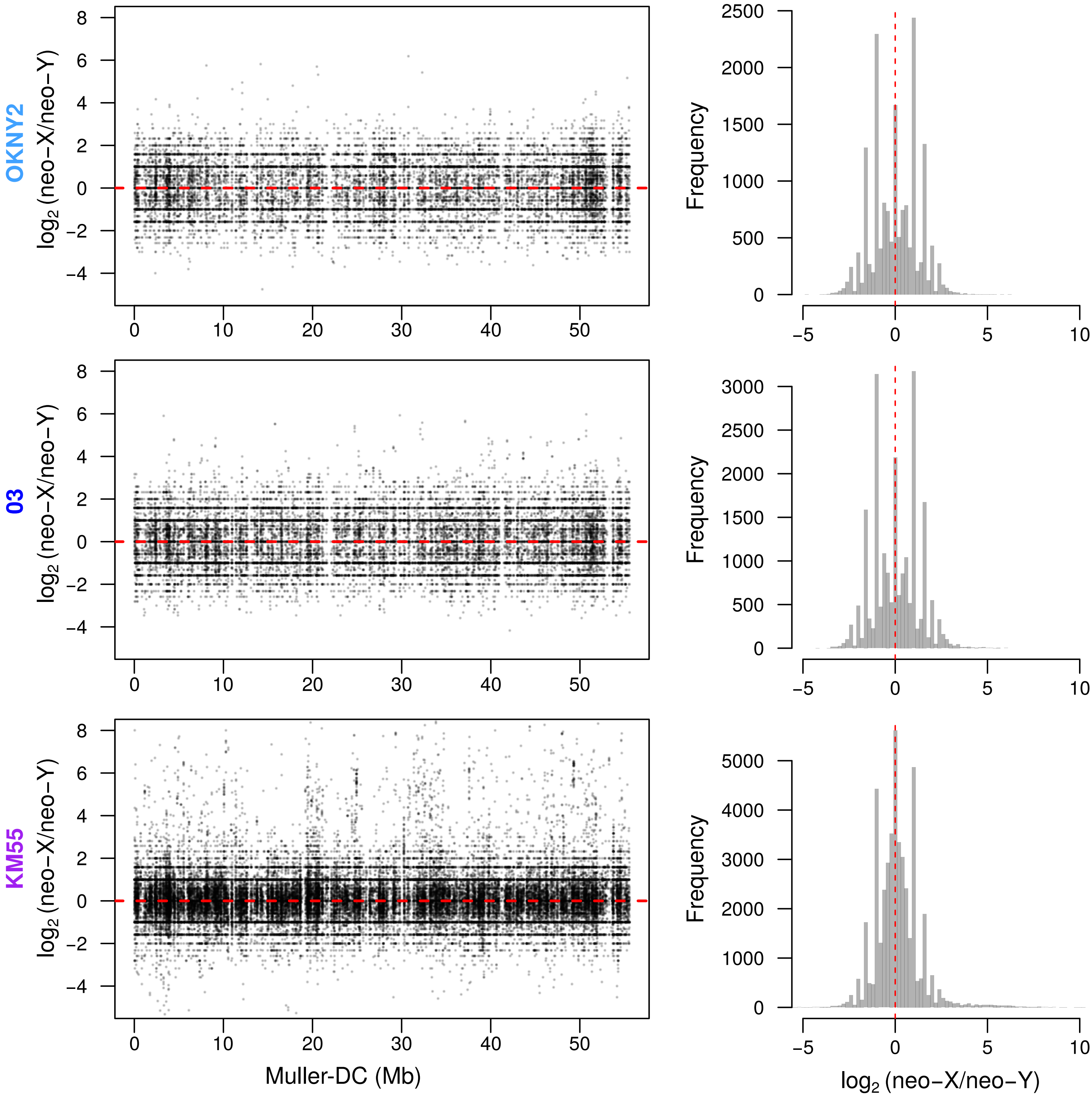

S9 Fig. Allele-specific differential expression at neo-X and neo-Y SNP sites. Left panels, allele-specific read counts over strain-specific SNP sites (points) differentiating the neo-X and neo-Y chromosomes were used to calculate the fold difference. Right panels, histograms of the distribution of the $\log_2$ fold differences. Red lines demarcate the median fold difference.
