# Supplementary material for: Ancestral male recombination in Drosophila albomicans produced geographically restricted neo-Y chromosome haplotypes varying in age and onset of decay
Source: PLoS Genet. 2019 Nov 18;15(11):e1008502. doi: 10.1371/journal.pgen.1008502 (PMC6897423; doi:10.1371/journal.pgen.1008502)
Supplement: S10 Fig — Neo-X and neo-Y reads were simulated at three different coverage ratios: 10x:5x (2-fold), 10x:8x (1.25-fold), and 12x:10x (1.2- fold). Fold-difference of allele specific read counts at each gene is plotted in log scale. Red dotted lines demarcate the median fold differences, and black dotted lines mark no expression difference. Across multiple levels allele-specific differences, our current pipeline is able to recapitulate the expected ratios, indicating that the lack of neo-X bias is not due to poor sensitivity in our pipeline. (PDF) [file pgen.1008502.s014.pdf]

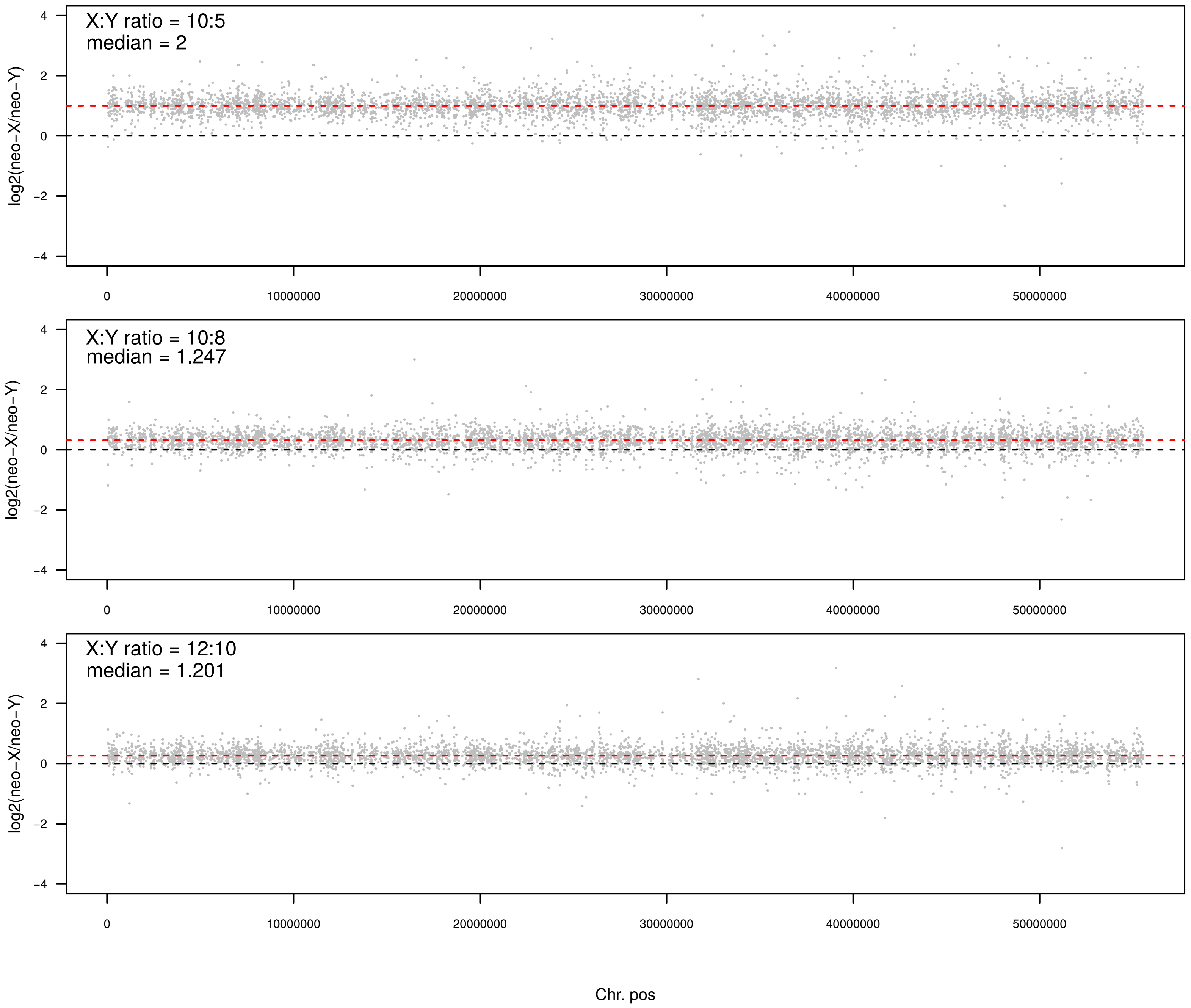

S10 Fig. Allele-specific expression on simulated KM55 data. Neo-X and neo-Y reads were simulated at three different coverage ratios: 10x:5x (2-fold), 10x:8x (1.25-fold), and 12x:10x (1.2- fold). Fold-difference of allele specific read counts at each gene are plotted in log scale. Red dotted lines demarcate the median fold differences, and black dotted lines mark no expression difference. Across multiple levels allele-specific differences, our current pipeline is able to recapitulate the expected ratios, indicating that the lack of neo-X bias is not due to poor sensitivity in our pipeline.
