# Supplementary material for: Ancestral male recombination in Drosophila albomicans produced geographically restricted neo-Y chromosome haplotypes varying in age and onset of decay
Source: PLoS Genet. 2019 Nov 18;15(11):e1008502. doi: 10.1371/journal.pgen.1008502 (PMC6897423; doi:10.1371/journal.pgen.1008502)
Supplement: S11 Fig — In Zhou and Bachtrog 2012, while the allele-specific expression for a large number of genes (n = 4839) were determined, only a small subset was used for the analysis (n = 805) after filtering out genes with fold differences (neo-X/neoY) greater than 1.25 or less than 0.75 in the male DNA. The purpose of this filter was to remove genes with substantial allele-bias at the DNA level, where the neo-X and neo-Y counts are expected to be highly similar. After reanalyzing the read count data generated by Zhou and Bachtrog 2012, the pipeline appears to produce extensive neo-X bias even from the DNA with a median fold difference of 1.56 (A); this is likely the result of reference allele bias, as the reference was generated from females, and therefore only contains the neo-X (also see S12 Fig). The allelic difference is further exaggerated in the RNA with median fold difference of 2.573. The filter therefore, at face value, seems like a sensible strategy to avoid genes with strong technical bias resulting from the pipeline. However, it substantially limited the number of genes being analyzed and reported, with only 16% of the genes being examined. This accounts for the large discrepancy between the number of genes examined between Zhou and Bachtrog 2012 and our study. In addition, Zhou and Bachtrog also attempted to correct for the bias by subtracting out the fold difference in the DNA from that of the RNA, reasoning that the reference allele bias should have similar effect for the DNA and RNA (panel B). Again at face value, this seems like a reasonable approach, but upon revisiting this correction, we do not think it is adequate. First the fold difference at the DNA level is positively but very poorly correlated with that of the RNA (R2 = 0.039, panel D). This argues that the former is a poor predictor of the latter. After the correction, the correlation becomes negative, with a equally poor R2 suggesting that the approach is performing poorly at correcting for the bias (pan [file pgen.1008502.s015.pdf]

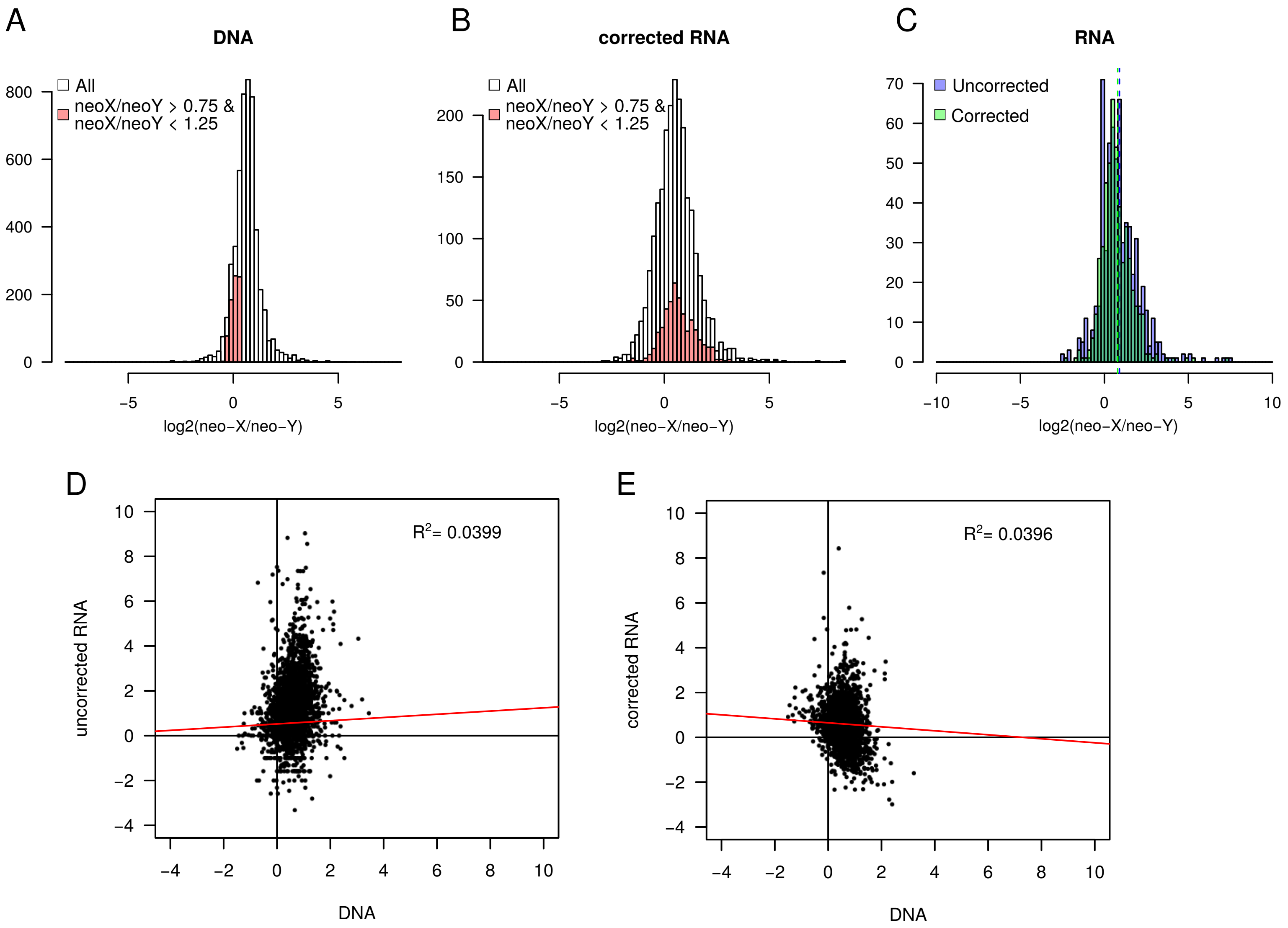

S11 Fig. Sources of discrepancy with Qi and Bachtrog 2012.

In Qi and Bachtrog 2012, while the allele-specific expression for a large number of genes (n = 4839) were determined, only a small subset was used for the analysis (n = 805) after filtering out genes with fold differences (neo-X/neoY) greater than 1.25 or less than 0.75 in the male DNA. The purpose of this filter was to remove genes with substantial allele-bias at the DNA level, where the neo-X and neo-Y counts are expected to be highly similar. After reanalyzing the read count data generated by Qi and Bachtrog 2012, the pipeline appears to produce extensive neo-X bias even from the DNA with a median fold difference of 1.56 (A); this is likely the result of reference allele bias, as the reference was generated from females, and therefore only contains the neo-X (also see supplementary figure 12). The allelic difference is further exaggerated in the RNA with median fold difference of 2.573. The filter therefore, at face value, seems like a sensible strategy to avoid genes with strong technical bias resulting from the pipeline. However, it substantially limited the number of genes being analyzed and reported, with only 16% of the genes being examined. This accounts for the large discrepancy between the number of genes examined between Qi and Bachtrog 2012 and our study.

In addition, Qi and Bachtrog also attempted to correct for the bias by subtracting out the fold difference in the DNA from that of the RNA, reasoning that the reference allele bias should have similar effect for the DNA and RNA (panel B). Again at face value, this seems like a reasonable approach, but upon revisting this correction, we do not think it is adequate. First the fold difference at the DNA level is postively but very poorly correlated with that of the RNA (R^2 = 0.039, panel D). This argues that the former is a poor predictor of the latter. After the correction, the correlation becomes negative, with a equally poor R^2 suggesting that the approach is performing poorly at correcting for the bias (panel E). The distribution of the fold difference at the DNA level is a combination of both the stochasticity in DNA amplification during library prep as well the technical biases introduced by the pipeline. The correction is implicitly assuming that only technical bias is contributing to the variance in the fold difference in the DNA and is to be subtracted from the RNA. This is also apparent when looking at the effect the correction has on the filtered genes where the correction has minimal effects on the fold difference of the filtered list of genes (panel C).

In short the pipeline used by Qi and Bachtrog introduced a substantial amount of reference allele bias that affected both the allele specific read counts in the male DNA and RNA and their approach of correcting for this was insufficient. The use of only one reference for allele-specific expression causes significant reference allele bias (see Stevenson, Coolon & Wittkopp 2013 and also supplementary figure 12). We therefore generated separate reference sequences for the neo-X and neo-Y. This substantially alleviated the neo-X bias as the median fold differences between the alleles across all male DNA samples are less than 1.05.
