# Supplementary material for: Ancestral male recombination in Drosophila albomicans produced geographically restricted neo-Y chromosome haplotypes varying in age and onset of decay
Source: PLoS Genet. 2019 Nov 18;15(11):e1008502. doi: 10.1371/journal.pgen.1008502 (PMC6897423; doi:10.1371/journal.pgen.1008502)
Supplement: S12 Fig — The high reference allele bias in Zhou & Bachtrog 2012 likely stem from the fact that reads derived from the neo-Y were only mapped to the neo-X, as the neo-Y was not assembled. In our current study, we generated line-specific "psuedoreferences" of both the neo-X and neo-Y, as to minimize reference allele bias. To demonstrate the extent to which reference allele bias affects allele-specific expression in this case, we, using the same DNA-seq and RNA-seq reads as Zhou & Bachtrog, first distinguished the neo-X and neo-Y reads by mapping to the two neo-sex pseudoreferences. We then remapped the neo-Y-mapping reads, to either the neo-X or neo-Y pseudo-references. This remapping was done using Tophat2 (instead of BWA like the rest of our study) to more closely emulate what was done by Zhou & Bachtrog 2012. After mapping to the neo-X reference, we compared the read counts of these neo-Y reads to the neo-X reads to infer allele-specific differences at genes. In both the DNA (panel A, blue) and RNA (panel B, blue) samples, we detected a significant bias for the neo-X allele (median log2 fold-change of 0.292, blue dotted line in Figure A), with the RNA sample showing stronger bias (median log2 fold-change of 0.460, blue dotted line in panel B). In contrast, when the same neo-Y reads were mapped to the neo-Y reference, the allele-specific differences are drastically reduced in both samples (red in panels A and B). Note, we are unable to recapitulate the severe extent of neo-X bias in both the DNA and RNA reported in Zhou & Bachtrog 2012 despite starting from the same raw reads. Given that an old version of Tophat was used (the no longer supported Tophat1), we suspect that more neo-Y reads failed to map, further exacerbating the reference/neo-X allele bias. (PDF) [file pgen.1008502.s016.pdf]

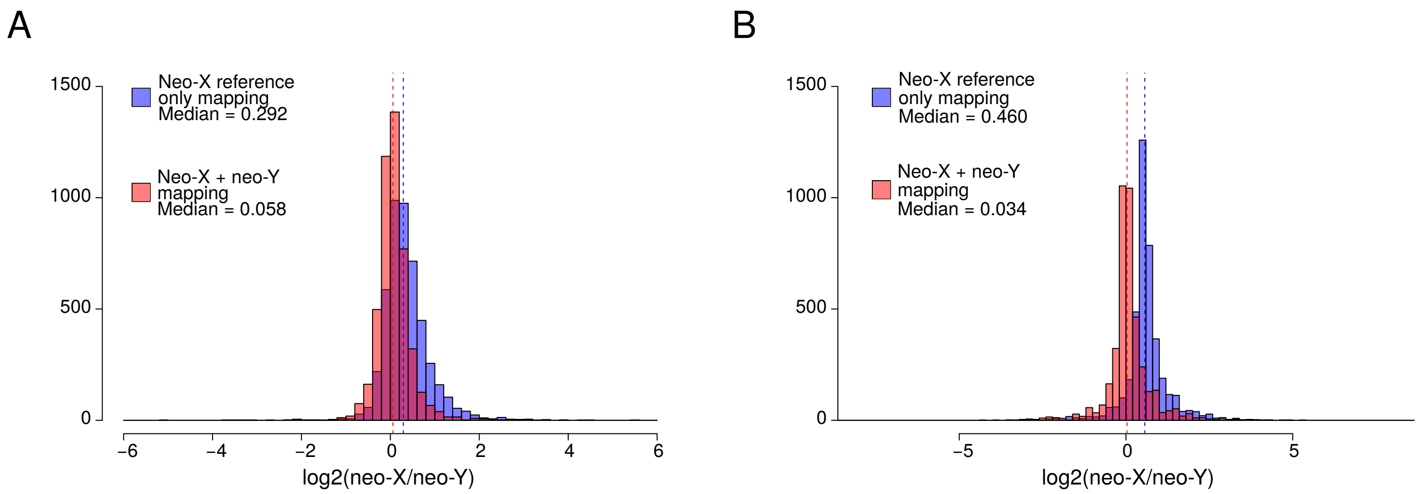

S12 Fig. Reference allele bias when mapping to only the neo-X chromosome.
The high reference allele bias in Qi & Bachtrog 2012 likely stem from the fact that reads derived from the neo-Y were only mapped to the neo-X, as the neo-Y was not assembled. In our current study, we generated line-specific "psuedoreferences" of both the neo-X and neo-Y, as to minimize reference allele bias. To demonstrate the extent to which reference allele bias affects allele-specific expression in this case, we, using the same DNA-seq and RNA-seq reads as Qi & Bachtrog, first distinguished the neo-X and neo-Y reads by mapping to the two neo-sex pseudoreferences. We then remapped the neo-Y-mapping reads, to either the neo-X or neo-Y pseudo-references. This remapping was done using Tophat2 (instead of BWA like the rest of our study) to more closely emulate what was done by Qi & Bachtrog 2012. After mapping to the neo-X reference, we compared the read counts of these neo-Y reads to the neo-X reads to infer allele-specific differences at genes. In both the DNA (panel A, blue) and RNA (panel B, blue) samples, we detected a significant bias for the neo-X allele (median $\log_2$ fold-change of 0.292, blue dotted line in Figure A), with the RNA sample showing stronger bias (median $\log_2$ fold-change of 0.460, blue dotted line in panel B). In contrast, when the same neo-Y reads were mapped to the neo-Y reference, the allele-specific differences are drastically reduced in both samples (red in panles A and B). Note, we are unable to recapitulate the severe extent of neo-X bias in both the DNA and RNA reported in Qi & Bachtrog 2012 despite starting from the same raw reads. Given that an old version of Tophat was used (the no longer supported Tophat1), we suspect that more neo-Y reads failed to map, further exacerbating the reference/neo-X allele bias.
