# Supplementary material for: Ancestral male recombination in Drosophila albomicans produced geographically restricted neo-Y chromosome haplotypes varying in age and onset of decay
Source: PLoS Genet. 2019 Nov 18;15(11):e1008502. doi: 10.1371/journal.pgen.1008502 (PMC6897423; doi:10.1371/journal.pgen.1008502)
Supplement: S13 Fig — Red lines demarcate the identify lines (x = y). (PDF) [file pgen.1008502.s017.pdf]

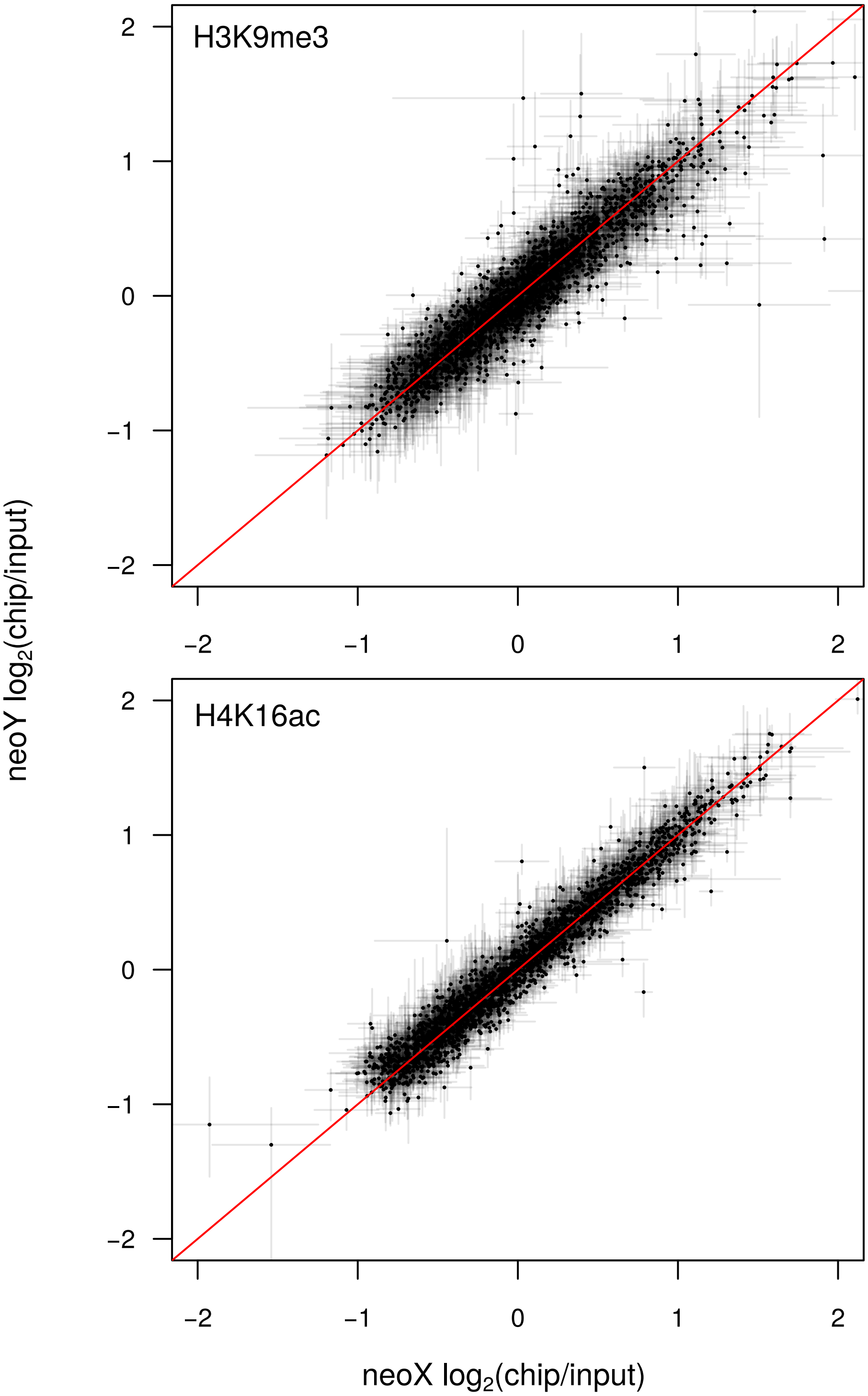

S13 Fig. Correlations of epigenetic mark enrichment on the neo-X and neo-Y. Red lines demaracate the identify lines (x=y)
