# Supplementary material for: Ancestral male recombination in Drosophila albomicans produced geographically restricted neo-Y chromosome haplotypes varying in age and onset of decay
Source: PLoS Genet. 2019 Nov 18;15(11):e1008502. doi: 10.1371/journal.pgen.1008502 (PMC6897423; doi:10.1371/journal.pgen.1008502)
Supplement: S14 Fig — Distribution of fixed derived SNPs (top) and indels (bottom) on the three neo-Y haplotypes: Y1 (blue), Y2 (light blue), Y3 (purple). (PDF) [file pgen.1008502.s018.pdf]

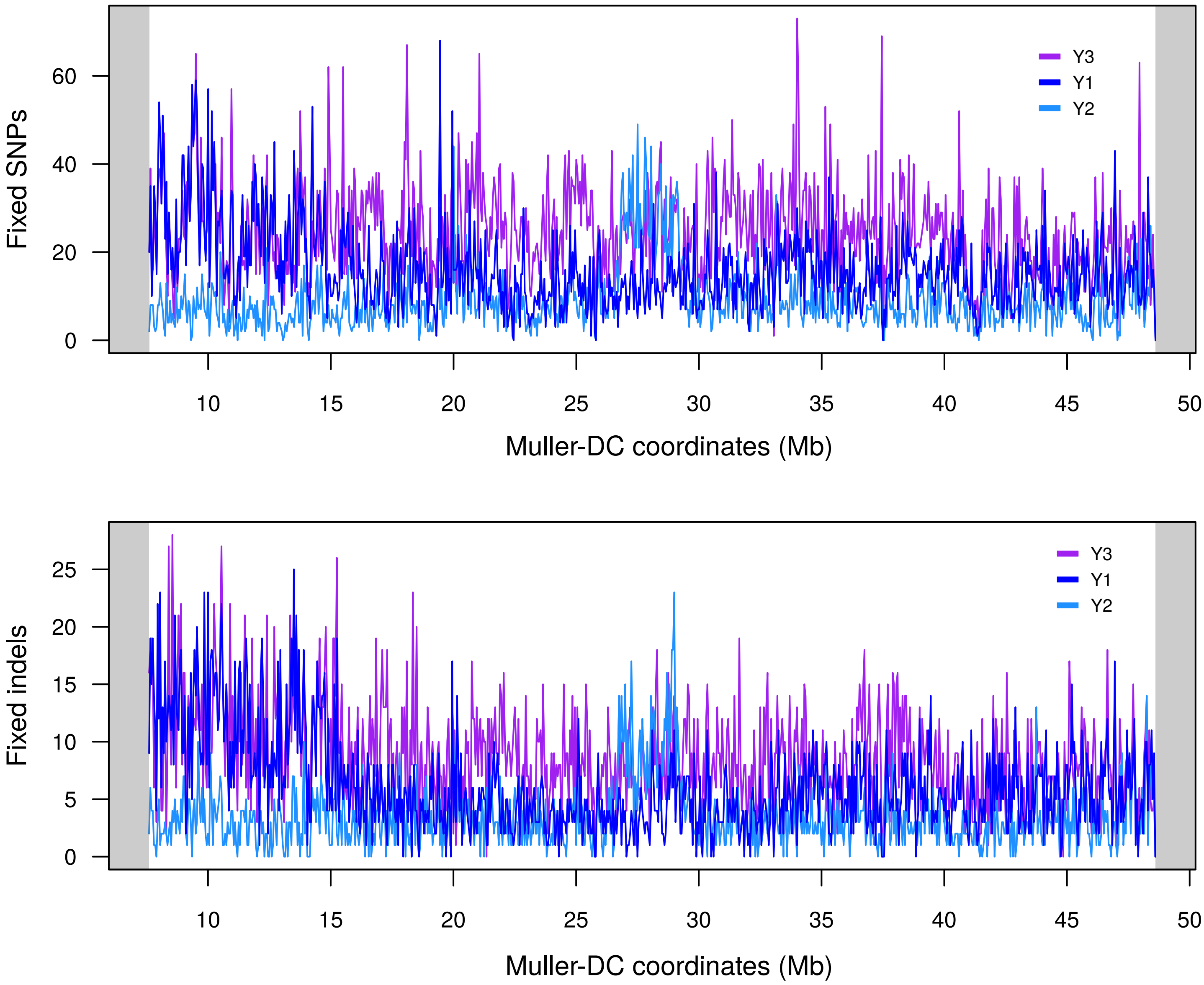

S14 Fig. Distribution of fixed derived SNPs (top) and indels (bottom) on the three neo-Y haplotypes: Y1 (blue), Y2 (light blue), Y3 (purple).
