# Supplementary material for: Ancestral male recombination in Drosophila albomicans produced geographically restricted neo-Y chromosome haplotypes varying in age and onset of decay
Source: PLoS Genet. 2019 Nov 18;15(11):e1008502. doi: 10.1371/journal.pgen.1008502 (PMC6897423; doi:10.1371/journal.pgen.1008502)
Supplement: Attachment — Submitted filename: Wei&Bachtrog_PLoSGenet_ms.docx [file pgen.1008502.s019.docx]

Study of Wei and Bachtrog investigated the unusual case of very young neo-Y chromosomes of *Drosophila albomicans*, which has ancestral male recombination but was suppressed across the male genome (achiasmy) after the neo-sex chromosome formed. The interesting combination of recombination dynamics in males, formation of neo-Y chromosomes, geographically isolated populations makes the study quite unique to address the unusual patterns of neo-Y evolution. Using genome-wide population genomic datasets, lab crosses and RNAseq gene expression datasets, the authors examined the molecular changes and evolutionary processes which initiated Y degeneration. They found evidences that recombination was suppressed at different time points across various populations in *D. albomicans* and as such the neo-Y haplotypes harbor different ages. In addition, they also found both coding and non-coding changes associates with ages of the neo-Y decay, in particular the fixed gene disruption via frameshift indels and down-regulation of new-Y alleles. They provided evidence that testes-expressed genes were shielded from degeneration at early stage.

I have read all previous three reviewers’ comments and the authors’ reply and changes/modifications in response to the comments. I think the authors have properly, and perhaps reasonably thoroughly addressed the vast majority of the concerns previously addressed.

I had pleasure reading this interesting work, which addresses the temporal dynamics in a short time window of neo-Y degeneration. I overall think the authors did a good job in presenting the complex story on various neo-Y haplotype recombination suppression and time course of neo-Y degeneration, and I do not have major criticisms on this manuscript or the work they reported. Nevertheless, there are several concerns or suggestions that might help improve the clarity and broader impact of this work.

1. In the Introduction and Discussion regarding the neo-Y chromosomes evolution or initial young sex chromosome degeneration, it could be more broader impact. The authors have almost exclusively focus on *Drosophila* taxa, perhaps only mention once japan sea stickleback work at some point. I think the authors could perhaps extend more on overall young or homomorphic sex chromosome systems. Would the molecular changes on initial sex chromosome degeneration occur in other young or homormophic sex chromosome systems, such as reptiles, amphibians, fish, even some plants? Also, there are more taxa showing (near) achiamy recombination systems, i.e. recent one in guppy, or some amphibians, which might share similar evolutionary trajectory of initial sex chromosome degeneration. I think this work does have broader impact, and the authors should add or extend a bit of this work in parallel to other young sex chromosome systems, better in the Introduction and Discussion sections.
2. I am a bit confused with the title. 1) Do the authors focus on neo-Y haplotypes in *Drosophila albomicans* and all other words are adjective to describe neo-Y? or they want to stress the correlation between neo-Y haplotypes and onset of decay? Perhaps try to make it less ambiguous. 2) Would it be better to change to ‘Ancestral male recombination’ instead of ‘Male recombination’? Currently, it reads as if they still occasionally recombine.
3. Previously one reviewer also mentioned, it would be more convenient if the authors could add line numbers throughout the text.
4. Page 3 top paragraph, to make easier understandable by broader readers (not sure people who do not work on sex chromosome field could immediately understand this), I think it is better to specify ‘In Drosophila males (XY), this is accomplished by chromosome-wide up-regulation of X-linked genes by epigenetic mechanisms’. Similarly, in the next sentence, ‘…on the Z chromosome of birds in females (ZW)’.
5. Page 3 second paragraph top sentence, ‘Unique to Drosophila and some other insects’, as previously mentioned, there are more taxa with (near) achiasmy being reported, so this sentence is better to be modified.
6. To be consistent in color code for neo-Y1, neo-Y2, neo-Y3 throughout the manuscript, please modify Fig. 2B the colors, Fig.3B and C, and also the relevant supplementary figures.
7. Figure 8 legend describes Pop. A and Pop. B, however, it was Pop.1 and Pop.2 in the figure itself, please be consistent in the labeling.
8. Page 12 the bottom paragraph top sentence (and also throughout the manuscript please check), the neo-X and neo-Y of *D. abomicans* are the youngest sex chromosomes investigated so far, not in the entire eukaryote taxa (there are much younger sex chromosomes in reptiles, amphibians, fish etc.), so here it should add ‘in Drosophila groups’.
9. Page 13 second paragraph, the comparison with Japan Sea Stickleback is not very clear, need to add a bit information on how old is the neo-Y in stickleback and the degeneration levels in this species.
