# Supplementary material for: Ancestral male recombination in Drosophila albomicans produced geographically restricted neo-Y chromosome haplotypes varying in age and onset of decay
Source: PLoS Genet. 2019 Nov 18;15(11):e1008502. doi: 10.1371/journal.pgen.1008502 (PMC6897423; doi:10.1371/journal.pgen.1008502)
Supplement: Attachment — Submitted filename: 1_ResponseToReviewers.docx [file pgen.1008502.s020.docx]

**Response to Reviewer #1:**

The authors have addressed most of my previous comments and they have done a considerable amount of additional work, including new tables and figures. I still have, however, some issues with the interpretation of the results shown in Table 2.

Table 2 (“both” column) shows that the fraction of genes (%) with either significant neo-X or neo-Y biased expression increases with the age of neo-Y haplotypes. However the next two columns show that the relative number of neo-X and neo-Y biased genes is maintained over time, which is difficult to explain under a simple model of gradual neo-Y degeneration. In my view, what we see is gene expression becoming more chromosome-specific (either neo-X or neo-Y biased but not both) with time. To me, this is perhaps an even more interesting observation. I feel that the manuscript would improve if this alternative interpretation were more clearly stated.

This is a very interesting point. We now also show expression changes (compared to *D. nasuta*) at neo-Y biased genes (Fig 7C), and find that neo-Y bias appears not to result from more expression from the neo-Y, but less expression from the neo-X. This patterns may refletct ‘demasculinization’ of the X, and we now discuss these results on page 11. However, we stress that given the small number of genes that are differentially expressed on Y1 and Y2 (the younger Y chromosomes), we are not entirely sure how to rigorously differentiate between the possibilities.

Suggested edits:

1. “Mai, Nalley and Bachtrog, in press” is the citation for the reference strain. Please include the full citation in the ‘References’ section.

Added the citation:

Dat Mai, Matthew J Nalley, Doris Bachtrog, Patterns of Genomic Differentiation in the Drosophila nasuta Species Complex, Molecular Biology and Evolution, msz215, <https://doi.org/10.1093/molbev/msz215>

2. “Together, these observations imply that the young neo-sex chromosomes of D. albomicans not only present a window to examine the initiating stages of sex chromosome differentiation”. “Initial” instead?

Changed.

3. “Shorter branch lengths indicate that the neo-Y haplotype ceased to recombine with the neo-X sooner after the formation of the species, and, extensively, an older neo-Y haplotype”. Perhaps “shortly after species formation” instead?

Changed the sentence to:

“Shorter branch lengths indicate that after the formation of the species, the neo-Y haplotype ceased to recombine with the neo-X earlier, and, extensively, an older neo-Y haplotype.”

4. “Monophyly of neo-Ys close to the centromere confirm that”. “confirms” instead?

Corrected.

5. “Based on the prevalent model whereby the neo-X and neo-Y fusions occurred after the species split [33-38], the two chromosomes are expected to be more similar to each other than to Chr. 3.” Which two chromosomes? Perhaps “...the two neo-sex chromosomes” instead?

Added.

6. “The distal end of Y3’s shorter D. nasuta haplotype block lands precisely at a haplotype breakpoint (Figure 3B and Figure 4D), arguing that male recombination truncated the segment with sequences from the neo-X.” The use of Y3’s is awkward. Perhaps “The distal end of the Y3 D. nasuta shorter haplotype” instead?

Changed to:

“The shorter D. nasuta haplotype block on Y3 has a distal end that lands precisely at a haplotype breakpoint”

7. “Interestingly, no neo-X biased genes are shared across all three neo-Ys and no more ...”. Neo-Ys is a bit awkward. Perhaps “neo-Y groups”?

Added.

**Response to Reviewer #2:**

This study uses genome-wide polymorphism and expression data to investigate patterns of neo-Y chromosome variation across different populations of Drosophila albomicans. The authors present evidence of multiple geographically restricted neo-Y haplotypes. While the nucleotide variation within each haplotype is limited, ancestral recombination between the neo-X and the neo-Y chromosomes has led to high genetic diversity between the neo-Y haplotypes. In addition, as recombination in the different populations was lost at different time points, there is evidence of incipient Y decay, the extent of degeneration correlating with the age of the neo-Y haplotypes. Overall, I found this manuscript to be very thorough and very well written. I only have a few comments and queries.

In the manuscript section relating to the correlation between neo-Y degeneration and haplotype age (page 10), the authors report that the number of non-synonymous mutations increases with age. While the oldest Y3 haplotype does indeed have the most overall substitutions within CDS and the youngest Y2 haplotype has the fewest, the proportion of non-synonymous substitutions out of the total number of substitutions is actually the lowest for the Y3 haplotype and the highest for the Y2 haplotype (Table 3). How do the authors explain this pattern?

This is a good point. We now also include Ka/Ks analysis across all genes, as the total number of amino-acid substitutions could be strongly influenced by a few outliner genes. Indeed, we find that Ka/Ks across all genes is significanly higher for the older Y haplotype, compared to the younger ones. This analysis is now presented as Figure 6D-F, and discussed in page 10.

Page 4: I understand that neo-Y sites were identified from neo-X sites that were homozygous in the females but heterozygous in the males. However, in the section “This generated 255,010 variant neo-Y sites across the eleven neo-Ys and 584,333 across the neo-sex chromosomes after filtering.”, I am a bit confused about where the difference between these two numbers of variant neo-Y sites comes from.

The former (neo-Y sites) are variant sites that differ between the neo-Ys i.e, polymorphic and fixed non-reference sites on the neo-Y, the latter are all the sites that differ between neo-Xs and neo-Ys, i.e. the variant neo-Y sites plus polymorphic neo-X sites. To clarify, we changed the sentence to:

“This generated 255,010 variant neo-Y sites across the eleven neo-Ys and 584,333 variant sites on both neo-sex chromosomes across all 28 strains after filtering.“

Page 6: “disruption of monophyly of the neo-Ys are due to recombination events with neo-Xs” – I think here it should be “is due to” instead of “are due to”.

Corrected.

Page 20 Figure 1 legend: “neo-Y samples collected from each site is labeled, and

color-coded based on grouping in C” - Here “is labeled” should be replaced with “are labeled”.

Corrected.

Figure 6: In this plot, I think it might be useful to add labels for each neo-Y haplotype as for example on page 10 there is mention of significant differences between Y1 and Y3 in Figure 6 yet there are no labels in this figure to point to the different haplotypes.

Added.

There is missing information about the sex of the d.alb.SHL48 and d.alb.SHL48_M individuals in Supplementary Table 1.

Thanks for catching this. Those were redundant/erroneous entries and are now removed. d.alb.SHL48 (female) is properly documented in a previous line as d.alb.E-10815_SHL48.

Throughout the manuscript, the authors should ensure consistent use of either “p-value” or “P-value”; “neo-X biased” or “neo-X-biased”; “Chr. 3” or “Chr3”.

Corrected.

**Response to Reviewer #3**:

Study of Wei and Bachtrog investigated the unusual case of very young neo-Y chromosomes of *Drosophila albomicans*, which has ancestral male recombination but was suppressed across the male genome (achiasmy) after the neo-sex chromosome formed. The interesting combination of recombination dynamics in males, formation of neo-Y chromosomes, geographically isolated populations makes the study quite unique to address the unusual patterns of neo-Y evolution. Using genome-wide population genomic datasets, lab crosses and RNAseq gene expression datasets, the authors examined the molecular changes and evolutionary processes which initiated Y degeneration. They found evidences that recombination was suppressed at different time points across various populations in *D. albomicans* and as such the neo-Y haplotypes harbor different ages. In addition, they also found both coding and non-coding changes associates with ages of the neo-Y decay, in particular the fixed gene disruption via frameshift indels and down-regulation of new-Y alleles. They provided evidence that testes-expressed genes were shielded from degeneration at early stage.

I have read all previous three reviewers’ comments and the authors’ reply and changes/modifications in response to the comments. I think the authors have properly, and perhaps reasonably thoroughly addressed the vast majority of the concerns previously addressed.

I had pleasure reading this interesting work, which addresses the temporal dynamics in a short time window of neo-Y degeneration. I overall think the authors did a good job in presenting the complex story on various neo-Y haplotype recombination suppression and time course of neo-Y degeneration, and I do not have major criticisms on this manuscript or the work they reported. Nevertheless, there are several concerns or suggestions that might help improve the clarity and broader impact of this work.

1. In the Introduction and Discussion regarding the neo-Y chromosomes evolution or initial young sex chromosome degeneration, it could be more broader impact. The authors have almost exclusively focus on *Drosophila* taxa, perhaps only mention once japan sea stickleback work at some point. I think the authors could perhaps extend more on overall young or homomorphic sex chromosome systems. Would the molecular changes on initial sex chromosome degeneration occur in other young or homormophic sex chromosome systems, such as reptiles, amphibians, fish, even some plants? Also, there are more taxa showing (near) achiamy recombination systems, i.e. recent one in guppy, or some amphibians, which might share similar evolutionary trajectory of initial sex chromosome degeneration. I think this work does have broader impact, and the authors should add or extend a bit of this work in parallel to other young sex chromosome systems, better in the Introduction and Discussion sections.

This is a good point, and we have broadened our introduction and discussion. In particular, we have added many more citations in the introduciton to non-Drosophila species when discussing general patterns of sex chromosome formation (page 2 & 3), and we have also included discussions and citations of other animal and plant systems when discussing the degeneration of young Y chromosomes in the Discussion section (page 13).

2. I am a bit confused with the title. 1) Do the authors focus on neo-Y haplotypes in *Drosophila albomicans* and all other words are adjective to describe neo-Y? or they want to stress the correlation between neo-Y haplotypes and onset of decay? Perhaps try to make it less ambiguous. 2) Would it be better to change to ‘Ancestral male recombination’ instead of ‘Male recombination’? Currently, it reads as if they still occasionally recombine.

Thank you for the suggestion. The title is indeed confusing (and wordy). We have changed it to “Ancestral male recombination in Drosophila albomicans produced geographically restricted neo-Y chromosome haplotypes varying in age and onset of decay”

3. Previously one reviewer also mentioned, it would be more convenient if the authors could add line numbers throughout the text.

Added.

4. Page 3 top paragraph, to make easier understandable by broader readers (not sure people who do not work on sex chromosome field could immediately understand this), I think it is better to specify ‘In Drosophila males (XY), this is accomplished by chromosome-wide up-regulation of X-linked genes by epigenetic mechanisms’. Similarly, in the next sentence, ‘…on the Z chromosome of birds in females (ZW)’.

Added.

5. Page 3 second paragraph top sentence, ‘Unique to Drosophila and some other insects’, as previously mentioned, there are more taxa with (near) achiasmy being reported, so this sentence is better to be modified.

Change to:

“Unlike most sexually reproduced organisms, Drosophila is peculiar since males are achiasmatic, that is, recombination does not occur during male meiosis.”

6. To be consistent in color code for neo-Y1, neo-Y2, neo-Y3 throughout the manuscript, please modify Fig. 2B the colors, Fig.3B and C, and also the relevant supplementary figures.

For 2B, using the three shades of blue/purple makes the figure difficult to look at because of the extensive overlaps between the lines. We, therefore, prefer to keep the color scheme as is for clarity. For 3B, we think that changing the color of the haplotype blocks may give the wrong impression that any of the haplotype block is the ancestral type. The colors were given based on the number of individuals having the same/monophyletic block. And since 3C is based on the haplotype blocks in 3B, we are keeping the same color scheme. We changed the colors of the labeling to be clearer. We also changed the labeling/color scheme in the supplementary figures accordingly.

7. Figure 8 legend describes Pop. A and Pop. B, however, it was Pop.1 and Pop.2 in the figure itself, please be consistent in the labeling.

Corrected. The label in the figure is changed to Pop.A and Pop.B.

8. Page 12 the bottom paragraph top sentence (and also throughout the manuscript please check), the neo-X and neo-Y of *D. abomicans* are the youngest sex chromosomes investigated so far, not in the entire eukaryote taxa (there are much younger sex chromosomes in reptiles, amphibians, fish etc.), so here it should add ‘in Drosophila groups’.

Changed the sentence to:

“To date, the neo-X and neo-Y of D. albomicans are *among* the youngest sex chromosomes to be investigated in detail. “

9. Page 13 second paragraph, the comparison with Japan Sea Stickleback is not very clear, need to add a bit information on how old is the neo-Y in stickleback and the degeneration levels in this species.

The age of the neo-Y and the level of degeneration are now added.
